# Postischemic Infusion of Apigenin Reduces Seizure Burden in Preterm Fetal Sheep

**DOI:** 10.3390/ijms242316926

**Published:** 2023-11-29

**Authors:** Kenta H. T. Cho, Natalya Hounsell, Evelyn McClendon, Art Riddle, Basappa, Simerdeep K. Dhillon, Laura Bennet, Stephen Back, Larry S. Sherman, Alistair J. Gunn, Justin M. Dean

**Affiliations:** 1 Department of Physiology, University of Auckland, Auckland 1142, New Zealand; kenta.cho@auckland.ac.nz (K.H.T.C.); nhou333@aucklanduni.ac.nz (N.H.); s.dhillon@auckland.ac.nz (S.K.D.); l.bennet@auckland.ac.nz (L.B.); aj.gunn@auckland.ac.nz (A.J.G.); 2 Department of Pediatrics, Oregon Health & Science University, Portland, OR 97239, USA; mcclende@ohsu.edu (E.M.); riddlea@ohsu.edu (A.R.); backs@ohsu.edu (S.B.); 3 Laboratory of Chemical Biology, Department of Studies in Organic Chemistry, University of Mysore, Manasagangotri, Mysore 570006, India; salundibasappa@gmail.com; 4 Department of Neurology, Oregon Health & Science University, Portland, OR 97239, USA; 5 Division of Neuroscience, Oregon National Primate Research Center, Beaverton, OR 97006, USA; shermanl@ohsu.edu; 6 Department of Cell, Developmental and Cancer Biology, Oregon Health & Science University, Portland, OR 97239, USA

**Keywords:** hypoxic–ischemic encephalopathy, seizures, anticonvulsants, apigenin, extracellular matrix, hyaluronidase, hyaluronidase inhibitor

## Abstract

Seizures are common in preterm newborns and are associated with poor neurodevelopmental outcomes. Current anticonvulsants have poor efficacy, and many have been associated with upregulation of apoptosis in the developing brain. Apigenin, a natural bioactive flavonoid, is a potent inhibitor of hyaluronidase and reduces seizures in adult animal models. However, its impact on perinatal seizures is unclear. In the present study, we examined the effect of apigenin and S3, a synthetic, selective hyaluronidase inhibitor, on seizures after cerebral ischemia in preterm fetal sheep at 0.7 gestation (98–99 days, term ~147 days). Fetuses received sham ischemia (*n* = 9) or ischemia induced by bilateral carotid occlusion for 25 min. Immediately after ischemia, fetuses received either a continuous infusion of vehicle (0.036% dimethyl sulfoxide, *n* = 8) or apigenin (50 µM, *n* = 6). In a pilot study, we also tested infusion of S3 (2 µM, *n* = 3). Fetuses were monitored continuously for 72 h after ischemia. Infusion of apigenin or S3 were both associated with reduced numbers of animals with seizures, total seizure time, and mean seizure burden. S3 was also associated with a reduction in the total number of seizures over the 72 h recovery period. In animals that developed seizures, apigenin was associated with earlier cessation of seizures. However, apigenin or S3 treatment did not alter recovery of electroencephalographic power or spectral edge frequency. These data support that targeting brain hyaluronidase activity with apigenin or S3 may be an effective strategy to reduce perinatal seizures following ischemia. Further studies are required to determine their effects on neurohistological outcomes.

## 1. Introduction

Perinatal hypoxic–ischemic (HI) brain injury is common in preterm infants, particularly before 28 weeks of gestation, and can lead to death, or in survivors, brain damage with lifelong disability [1,2]. Both clinical and experimental studies have shown that hypoxic–ischemic encephalopathy (HIE) is not a single event, but rather an evolving process leading to the clinical manifestation of brain injury, such as cell death and seizures, many hours after the initial insult [3,4,5].

Seizures develop in over 60% of neonates with HIE and are the most common neurological emergency in infants [6,7]. While seizures in the setting of HIE are closely associated with poor neurodevelopmental outcomes, the degree to which seizures reflect underlying brain injury or modulate the evolution of injury is unclear [4,8]. Despite this uncertainty, there is evidence from human and preclinical animal studies of HI that seizures may independently worsen brain injury [6,8,9]. Clinically, there is evidence that seizures increase cerebral metabolism [10] and are closely associated with neuronal loss on modern imaging [11]. Additionally, seizures can promote abnormal synaptic reorganization and increase the risk for later development of epilepsy [12,13]. However, the most effective approach to managing neonatal seizures is unclear because of the poor efficacy of current anticonvulsants and the association of anticonvulsants with adverse effects on the developing brain [14,15,16]. Therefore, there is an urgent need for further treatment strategies to reduce the seizure burden associated with perinatal HI.

The mechanisms underlying seizure development are complex and not fully understood. There is increasing evidence of a role for disruption of the brain’s extracellular matrix (ECM). In in vitro and in vivo studies, enzymatic degradation [17,18] or knockout [19,20] of brain hyaluronan (the major component of the ECM) was associated with epileptogenesis. Further, disruption of brain hyaluronan was observed in status epilepticus in adult mice [21] and in human temporal lobe epilepsy [22]. Although it is unclear whether ECM disruption plays a direct causative role in seizures in the developing brain, we and others have reported abnormal upregulation of brain hyaluronidase enzymes [23,24] and disruption of the hyaluronan-based ECM [23,25,26] following HI in fetal sheep and neonatal rodents.

Apigenin is a natural bioactive flavonoid present in common fruits and vegetables, and possesses a wide range of pharmacological properties, including inhibition of hyaluronidase activity [27,28,29,30,31]. Interestingly, prophylactic apigenin treatment was reported to reduce seizure severity and improve seizure-related outcomes in adult animal models of chemical-induced epilepsy [32,33,34]. Further, treatment with the natural apigenin derivative vitexin (apigenin-8-C-glucoside) immediately after HI in postnatal day (P)7 rats was associated with reduced spontaneous seizures at P31 [35]. Despite these findings, the effect of apigenin on development of seizures and recovery of brain activity after acute HI in the developing brain is unknown.

Therefore, in the present study, we examined the effect of apigenin treatment on neurophysiological recovery after acute ischemia induced by bilateral carotid occlusion in preterm fetal sheep at 0.7 of gestation. At this gestational age, the neural maturation of fetal sheep is broadly equivalent to 28–32 weeks of human development [36]. In a preliminary study, we then examined the hypothesis that hyaluronidase activity has a key role in seizure activity following ischemia in the 0.7 gestation sheep by contrasting the effects of apigenin with S3, an apigenin-based drug whose structure is optimized to selectively inhibit hyaluronidase activity [37].

## 2. Results

### 2.1. Fetal Biochemistry and Postmortem Data

All ischemia animals received a complete 25 min period of bilateral carotid artery occlusion. There were no significant differences in pH, partial pressure of carbon dioxide (PaCO_2_), partial pressure of oxygen (PaO_2_), glucose, or lactate levels between the ischemia-apigenin or ischemia S3 groups and the ischemic vehicle group in the baseline period (Table 1). Following ischemia, glucose levels in the ischemia vehicle group was significantly elevated at 10 min recovery (*p* = 0.032 vs. sham ischemia), and lactate levels were increased at 10 min (*p* = 0.011 vs. sham ischemia) and 30 min (*p* = 0.048 vs. sham ischemia) recovery, while there were no changes in any other parameters. There were no significant differences in any of the parameters between the ischemia vehicle group and the ischemia apigenin or ischemia S3 groups at any recovery timepoint. At postmortem, there were no significant differences in fetal sex or fetal body weights between the groups (Table 2). Fetal brain weight was reduced in the ischemia vehicle, ischemia apigenin, and ischemia S3 groups compared with sham ischemia (*p* = 0.036 vs. ischemia vehicle, *p* = 0.007 vs. ischemia apigenin, *p* = 0.002 vs. ischemia S3), but not different between the ischemia groups. 

### 2.2. Electroencephalographic (EEG) Power, Spectral Edge Frequency (SEF), and EEG Frequency Bands

**Figure 1:**
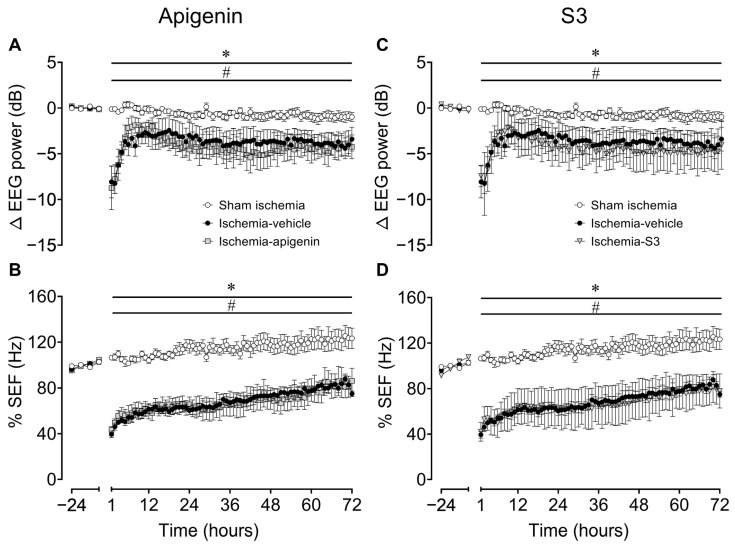
Time sequence of changes in fetal electroencephalographic power (∆EEG, dB) (**A**,**C**) and percentage changes in spectral edge frequency (%SEF, Hz) (**B**,**D**) from 24 h before until 72 h after cerebral ischemia induced by 25 min bilateral carotid occlusion in the sham ischemia (open circles; *n* = 9; 4 females, 5 males), ischemia vehicle (closed circles; *n* = 8; 3 females, 5 males), ischemia apigenin ((**A**,**B**); grey squares; *n* = 6, 4 females, 2 males), and ischemia S3 ((**C**,**D**); grey triangles; *n* = 3, 2 females, 1 male) groups. Time zero denotes the end of the period of 25 min of cerebral ischemia. Note that for presentation purposes, EEG and SEF datasets for ischemia apigenin and ischemia S3 are plotted as separate graphs but contain the same sham ischemia and ischemia vehicle datasets. Data are mean ± standard error of the mean (SEM) (of 6 h averages for baseline period and 1 h averages post-occlusion). Statistical significance was determined using repeated measures analysis of variance (ANOVA), followed by Fisher’s least significant difference (LSD) post-hoc analysis. * *p* < 0.05, sham ischemia vs. ischemia vehicle; ^#^ *p* < 0.05, sham ischemia vs. ischemia apigenin or ischemia S3.

There were no significant differences in electroencephalographic (EEG) power (Figure 1A,C) or spectral edge frequency (SEF; Figure 1B,D) between groups in the baseline period. Cerebral ischemia was associated with rapid, profound suppression of EEG power (last minute of ischemia: sham ischemia 18.0 ± 1.2 dB vs. ischemia-vehicle 9.8 ± 1.3 dB, *p* = 0.01; ischemia apigenin 7.0 ± 3.4 dB, *p* = 0.002; ischemia S3 5.0 ± 2.6 dB, *p* = 0.001) and SEF (last minute of ischemia: sham ischemia 9.5 ± 0.8 Hz vs. ischemia vehicle 2.9 ± 0.3 Hz, *p* < 0.0001; ischemia apigenin 4.3 ± 0.8 Hz, *p* = 0.007; ischemia S3 5.7 ± 2.6 Hz; *p* = 0.004), which did not differ between the ischemia groups. Post-ischemia, both EEG and SEF were significantly suppressed in all ischemia groups for the entire 72 h period (EEG: *p* = 0.031, sham ischemia vs. ischemia vehicle; *p* = 0.022, sham ischemia vs. ischemia apigenin; *p* = 0.049, ischemia S3 vs. sham ischemia; SEF: *p* < 0.001, sham ischemia vs. ischemia vehicle; *p* < 0.001, sham ischemia vs. ischemia apigenin; *p* = 0.005, sham ischemia vs. ischemia S3), with no significant differences between the ischemia groups. For presentation purposes, EEG and SEF datasets for ischemia apigenin (Figure 1A,B) and ischemia S3 (Figure 1C,D) are plotted separately. 

EEG power was also analyzed in individual EEG frequency bands. There were no significant differences in any EEG frequency band between groups in the baseline period (Figures S1 and S2). After ischemia, relative delta frequency was greater, while the relative theta, alpha, and beta frequencies were less after ischemia vehicle, ischemia apigenin (Figure S1A–D), and ischemia S3 (Figure S2A–D; except for the theta band, which only showed a trend towards being lower) for the entire 72 h recovery period compared with the sham ischemia group (*p* = 0.001 vs. ischemia vehicle, *p* = 0.006 vs. ischemia apigenin, *p* = 0.030 vs. ischemia S3). There were no significant differences between the ischemia groups.

### 2.3. Seizure Activity with Apigenin Treatment

**Figure 2:**
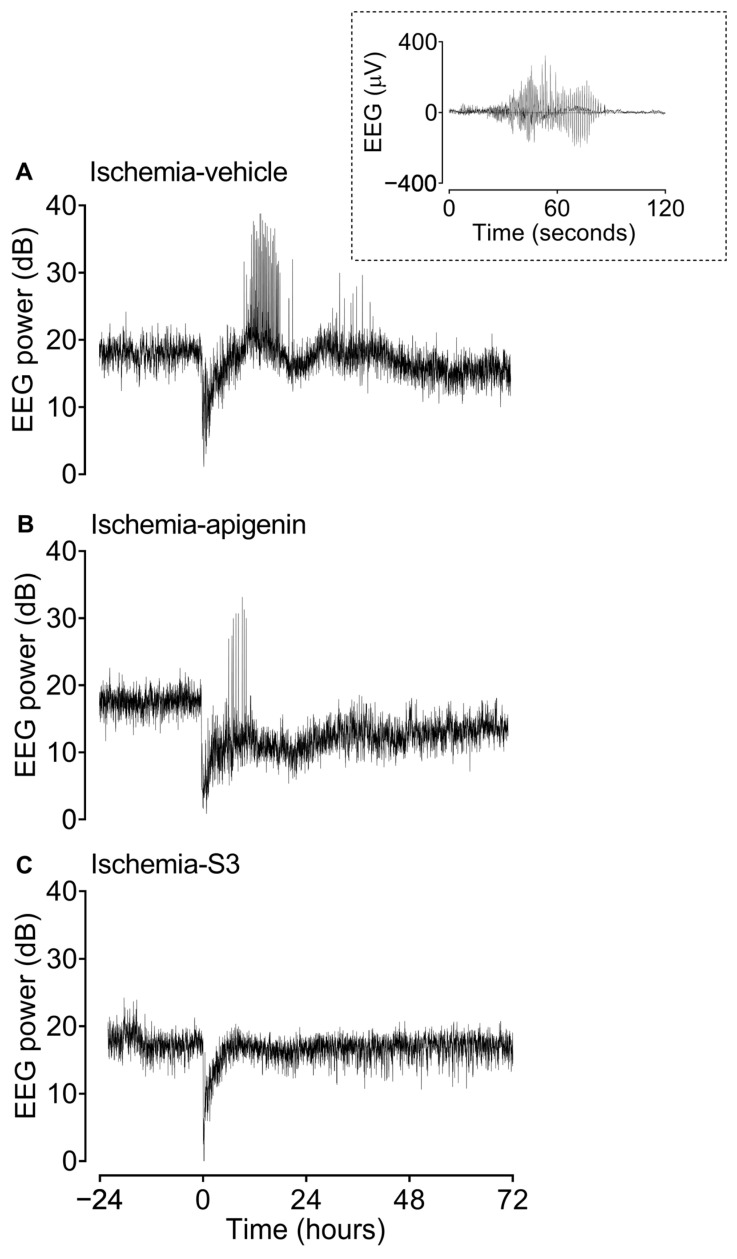
Examples of continuous 1 min average EEG power data from fetuses in the ischemia vehicle (**A**), ischemia apigenin (**B**), and ischemia S3 (**C**) groups before, during, and after cerebral ischemia. Apigenin or S3 was infused immediately following ischemia for 24 h. (**A**) Representative ischemia vehicle group animal showing suppression of EEG power after cerebral ischemia (0 h), followed by high-amplitude EEG events between ~5 h and 48 h, reflecting electrographic seizures. (**B**) Representative ischemia apigenin group animal showing a discrete period of seizures between ~5 h and 11 h. (**C**) Representative ischemia S3 group animal showing profound suppression of post-ischemic seizures. Inset: example of a stereotypic evolving seizure from a fetus in the ischemia vehicle group. Seizures were defined as the concurrent appearance of sudden, repetitive, and rhythmic waveforms in the EEG signal lasting >10 s with a stereotypic evolving nature, a minimum seizure amplitude of 20 μV [38], and a minimum seizure amplitude of 20 μV. Note that stereotypic evolving seizures were seen in the ischemia vehicle, ischemia apigenin, and ischemia S3 groups.

**Figure 3:**
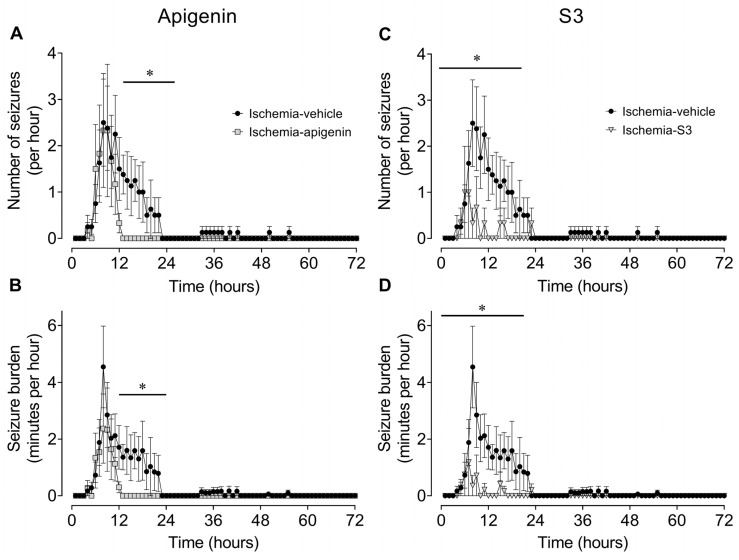
Time sequence of changes in fetal stereotypic evolving seizures after cerebral ischemia. (**A**,**C**) The numbers of seizures per hour in the ischemia vehicle (closed circles; *n* = 8; 3 females, 5 males), ischemia-apigenin ((**A**); grey squares; *n* = 6; 4 females, 2 males), and ischemia-S3 ((**C**); grey triangles; *n* = 3; 2 females, 1 male) groups. (**B**,**D**) The total seizure burden per hour (i.e., minutes seizing per hour) in the ischemia vehicle (closed circles; *n* = 8; 3 females, 5 males), ischemia apigenin ((**B**); grey squares; *n* = 6; 3 females, 3 males), and ischemia S3 ((**D**); grey triangles; *n* = 3; 2 females, 1 male) groups. Data are mean ± SEM. Statistical significance was determined using repeated measures ANOVA, followed by Fisher’s LSD post-hoc analysis. * *p* < 0.05, vs. ischemia vehicle.

Details of the various seizure parameters used are defined in Section 4.6. Representative examples of a stereotypic evolving seizure and 1 min average EEG data from fetuses in the various groups are shown in Figure 2. No seizures were seen in the sham ischemia group. Compared with the ischemia vehicle group, apigenin treatment was associated with a significant reduction in the number of animals that developed seizures (*p* = 0.020; Table 3), total seizure time (*p* = 0.029; Table 3), but no significant effect on numbers of seizures over the 72 h recovery period (*p* = 0.073; Table 3). In animals that developed seizures, apigenin treatment was also associated with earlier resolution of seizures (i.e., seizure end, *p* = 0.024 vs. ischemia vehicle; Table 3), but there was no significant change in average seizure duration (*p* = 0.084 vs. ischemia vehicle; Table 3). Finally, apigenin treatment was associated with a significant reduction in the mean number of seizures per hour (*p* = 0.016 vs. ischemia vehicle; Figure 3A) and in mean seizure burden (i.e., seizure minutes per hour, *p* = 0.025 vs. ischemia vehicle; Figure 3B) from 12 to 24 h post-ischemia.

### 2.4. Seizure Activity with S3 Treatment

Compared with ischemia vehicle, treatment with S3 [37] was associated with a significant reduction in numbers of animals developing seizures (*p* = 0.010; Table 3), total seizure time (*p* = 0.048; Table 3), and total number of seizures over the 72 h recovery period (*p* = 0.030; Table 3). S3 treatment was also associated with a significant reduction in the mean number of seizures per hour (*p* = 0.023 vs. ischemia vehicle; Figure 3C) and the mean seizure burden (i.e., seizure minutes per hour, *p* = 0.006 vs. ischemia vehicle; Figure 3D) at 6–24 h post-ischemia (earlier than after apigenin treatment). 

### 2.5. Fetal Arterial Blood Pressure and Heart Rate

**Figure 4:**
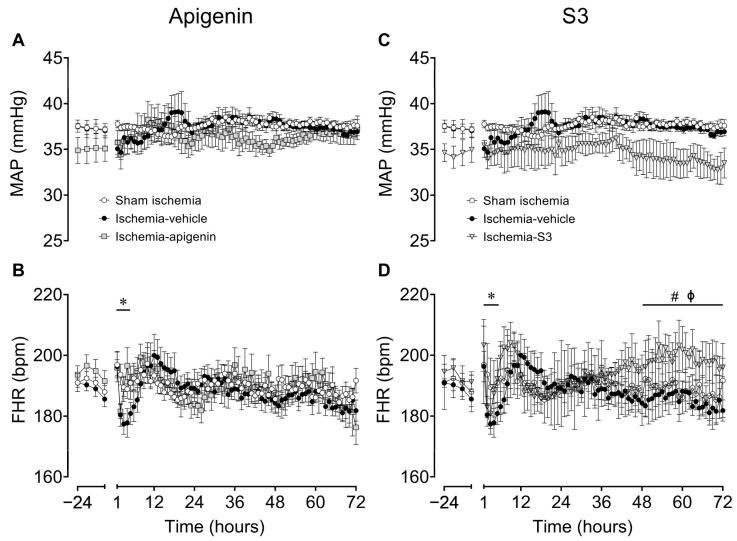
Time sequence of changes in fetal mean arterial pressure (MAP; (**A**,**C**)) and fetal heart rate (FHR; (**B**,**D**)) from 24 h before until 72 h after cerebral ischemia induced by 25 min of bilateral carotid occlusion in the sham ischemia (open circles; *n* = 9; 4 females, 5 males), ischemia vehicle (closed circles; *n* = 8; 3 females, 5 males), ischemia apigenin (grey squares; *n* = 6; 4 females, 2 males), and ischemia S3 ((**C**), grey triangles; *n* = 3; 2 females, 1 male) groups. Time zero denotes the end of the period of 25 min of cerebral ischemia. Data are means  ±  SEM (of 6 h averages for baseline period and 1 h averages post-occlusion). Statistical significance for FHR was determined using repeated measures ANOVA, followed by Fisher’s LSD post-hoc analysis. Statistical significance for MAP was determined using normalized data because of differences between groups at baseline and analyzed using repeated measures ANOVA, followed by Fisher’s LSD post-hoc analysis. * *p* < 0.05, sham ischemia vs. ischemia vehicle; ^#^ *p* < 0.05, sham ischemia vs. ischemia apigenin or ischemia-S3; ^ϕ^ *p* < 0.05, ischemia vehicle vs. ischemia apigenin or ischemia S3.

In all ischemia animals, carotid artery occlusion was associated with a significant increase in mean arterial blood pressure (MAP) and fetal heart rate (FHR) during the occlusion period. Following the end of occlusion, the ischemia vehicle group showed a statistically borderline, transient fall in MAP hypotension (*p* = 0.051 vs. sham ischemia; Figure 4A) and reduced FHR (*p* = 0.039 vs. sham ischemia; Figure 3B) over the first 6 h of recovery. Note that this fall in FHR was not as prominent in the ischemia apigenin and ischemia S3 groups. Nevertheless, there were no significant differences in MAP between the ischemia vehicle group and the ischemia apigenin or ischemia S3 groups at any time during the recovery period. Similarly, there was no significant difference in FHR between the ischemia vehicle group and the ischemia apigenin group. However, S3 treatment was associated with a mild increase in FHR between 48 and 72 h post-ischemia (*p* = 0.026 vs. sham ischemia and ischemia vehicle groups).

## 3. Discussion

The present study demonstrates that two flavonoid-derived compounds, apigenin and S3, can reduce the incidence of fetal seizures when administered after severe cerebral ischemia. Post-ischemic infusion of apigenin was associated with reduced numbers of animals developing seizures, total seizure burden (min), numbers of seizures per hour (from 12 to 24 h post-ischemia), and mean seizure burden (i.e., seizure minutes per hour; from 12 to 24 h post-ischemia), as well as earlier cessation of seizures. Although apigenin has multiple potential mechanisms of actions [28,39], in a preliminary study, we found that the highly selective hyaluronidase inhibitor S3 was associated with a similar profile of antiseizure effects to apigenin. Thus, these findings support a potential role for hyaluronidases enzymes in regulating seizure activity after perinatal cerebral ischemia and provide proof-of-principle evidence that targeting hyaluronidase activity may be an effective strategy for reducing perinatal seizures.

It is now well established that brain injury evolves in distinct phases after transient HI [3,4]. Critically, in the so-called latent phase after reperfusion, there can be transient recovery of mitochondrial activity and oxidative metabolism for up to 6 h, despite ongoing suppression of EEG activity [3]. This is followed by a delayed deterioration over approximately 72 h in a secondary phase, as shown by loss of oxidative metabolism, cytotoxic edema, and delayed onset of seizures [3]. Given the delayed onset of seizures and major neurodegeneration occurring in this secondary phase, therapies commenced in the preceding latent phase may improve outcomes, and so offer a potential window of therapeutic opportunity. In the present study, we initiated apigenin and S3 treatments immediately after the end of ischemia to establish proof-of-principle efficacy. Future studies are needed to evaluate the therapeutic window. In the present study, all animals in the ischemia vehicle group developed seizures after cerebral ischemia. Overt, stereotypic seizures began at a mean of 7.0 ± 1.0 h of recovery, with maximal seizure activity (as measured by numbers of seizures and seizure burden) peaking at 8–11 h post-ischemia, which then largely resolved by 23 h of recovery. 

These data are broadly consistent with the recent clinical evidence observed from the HELIX trial in which seizures developed from 1.4–5 h postpartum in normothermic term-infants, with a median seizure period of 19 h (defined as the time between first and last seizure) [40]. Other studies in term infants have observed seizure onset times from 10 to 15 h following intrapartum HI, and maximal seizure activity in the first 24 h after HI [6,41]. In comparison, longer latency of seizure onset has been reported in preterm infants, with identification of the first seizure days following the insult [42,43,44,45]. This may, in part, be due to the lack of a systematic approach to initiating EEG monitoring early in preterm neonates [45]. Despite the evidence that exclusively subclinical seizures occur more frequently than in term infants [43], there is significant lack of understanding of the temporal evolution of preterm seizures. Furthermore, the late onset of seizures in preterm neonates may also relate to the underlying pathophysiology, where seizures resulting from other common preterm etiologies may develop later [44,45,46].

In the present study, in contrast with the vehicle controls, only 50% of animals developed seizures after post-ischemic apigenin treatment, and those that did develop seizures had markedly reduced overall seizure burden and earlier resolution of seizures. Similarly, infusion of the selective hyaluronidase inhibitor S3 was also associated with antiseizure effects, with only one of three treated animals having seizures, and reduced total seizure time and numbers of seizures over the 72 h recovery period. These findings are consistent with evidence that flavonoids, including apigenin, have anticonvulsant effects in multiple animal models of epilepsy [32,33,47].

The specific mechanisms of seizure suppression with apigenin and S3 treatment in the present study are unclear. Nevertheless, both apigenin and S3 are well-known hyaluronidase inhibitors that block enzymatic digestion of hyaluronan, a key component of the brain extracellular matrix [17,31,37]. Experimentally, dysregulation of brain hyaluronan (via hyaluronidase activity) is closely linked with increased neuronal excitability and epileptogenesis [17,20,21]. In preterm fetal sheep, we have previously shown increased expression of brain hyaluronidases from 1 day to 4 weeks following cerebral HI [24]. Similarly, persistent increases in brain hyaluronidase expression were reported from early as 1 h post-ischemia in adult rodents [48,49] and from 24 h following HI in neonatal rodents [23]. Overall, these studies support a role for abnormal hyaluronidase activity in regulating seizure development after perinatal cerebral ischemia and suggest that treatment with apigenin or S3 may modulate seizure via targeting hyaluronidase activity. However, future studies are required to determine the temporal relationship between hyaluronidase activity, dysregulation of the brain extracellular matrix, and seizures following cerebral ischemia in preterm fetal sheep. 

Apigenin treatment has also been shown to confer neuroprotection against HI brain injury in neonatal rats [50], while S3 accelerated oligodendrocyte maturation and promotes myelination in an in vitro model of perinatal white matter injury [37]. Thus, the reduction in seizures in the apigenin- or S3-treated animals in the present study may reflect reduced cellular injury. There is also evidence of significant crosstalk between the brain ECM and neuronal survival following HI [25,26]. For example, degradation of the brain ECM can increase oxidative stress and cause death of neuronal subtypes, including GABAergic interneurons that regulate neural excitation and seizure activity [51,52,53]. However, the degree to which seizure activity reflects underlying neural injury or modulates the evolution of injury is unclear [4], and whether the antiseizure effects seen the present study are associated with improved neurohistological outcomes requires investigation. 

Alternatively, other biological actions of apigenin may contribute to its anticonvulsant actions, including anti-excitatory, antioxidant, and anti-inflammatory effects [28]. For example, apigenin can reduce glutamate receptor activation in cultured neurons and protect against glutamate-induced neurotoxicity [54,55]. Further, apigenin can inhibit glutamate-mediated effects via increased intracellular levels of the antioxidant reduced glutathione [32]. There may also be a role for apigenin in modulating GABAergic neurotransmission via upregulation of GABA levels [47]. However, the effect of apigenin on GABA receptors remains unclear, as it appears to have a range of GABAergic actions, including as an antagonist [55,56] and a potential agonist [57]. Future studies examining the effect of apigenin (and S3) on glutamate and GABA receptors in fetal sheep are required. Nevertheless, we previously reported in fetal sheep that treatment with specific glutamate receptor antagonists, which directly exert neural inhibitory effects, are associated with immediate EEG suppression and antiseizure effects after HI [58,59]. By contrast, in the present study, treatment with apigenin or S3 did not significantly modulate EEG power or frequency during the period of drug infusion or post-infusion recovery, even though treatment was started immediately after the end of ischemia. Overall, these findings support that the seizure suppression with apigenin and S3 treatment in the present study was likely mediated by modulation of brain ECM disruption and brain injury associated with HI, rather than a primary inhibition of neural activity. 

Notably, while apigenin and S3 treatments did not improve EEG recovery over 3 days after HI in the present study, both clinical and experimental studies have shown that EEG recovery after injury does not accurately reflect outcomes [3]. Further, we previously reported a nonlinear relationship between EEG power and neuronal survival [60]. Nevertheless, further long-term recovery studies are required to comprehensively assess the relationship between seizures, hyaluronidase activity, and functional outcomes based on EEG.

In the present study, infusion of apigenin after recovery from cerebral ischemia was not associated with any systemic cardiovascular effects. This is consistent with the finding in normotensive rats that central administration of apigenin did not alter blood pressure or heart rate [61]. Intriguingly, apigenin was reported to have significant hypotensive effects in models of hypertension by reducing vascular reactivity to vasoconstrictors, oxidative stress, and the production of vasopressors such as angiotensin II [61,62,63]. In future studies, it may be important to assess the cardiovascular (and antiseizure) actions of apigenin following other insults known to cause perinatal HIE, such global hypoxia, which is associated with post-asphyxial hypertension and hemodynamic instability [64]. By contrast, S3 was associated with a mildly increased FHR following the end of drug infusion from 48 to 72 h post-ischemia, without changes in blood pressure. The mechanism mediating this is unclear—potentially, this could reflect a direct physiological effect of S3 or an adaptation to the end of S3 infusion.

An important consideration for future studies is that the optimal doses of apigenin and S3 are unclear. There is evidence that apigenin has a concentration-dependent response, with higher concentrations providing a more rapid and marked anticonvulsant effect in mice [32]. Thus, future drug dose–response studies are required. Equally, future pragmatic studies focusing on minimally invasive routes of administration (e.g., intranasal or intravenous), delayed treatment onset (including after seizure onset), and comparisons with common anticonvulsants (e.g., phenobarbital and levetiracetam) are critical for potential translation. While the preterm fetal sheep is a highly clinically translatable model of HIE, developmental expression of brain hyaluronidases and the impact of hyaluronidase inhibitors in the human neonate remain largely unknown. Human neuropathological studies are needed to examine hyaluronidase expression and the potential impact of HI. Finally, a potential limitation of our data is the small number of animals that developed seizures in the treatment groups, making it difficult to interpret apparent changes in seizure characteristics. 

In conclusion, the present study demonstrated that post-ischemic administration of apigenin or S3 significantly reduced seizures in the fetal sheep. These findings highlight a potential role for disruption of the ECM in regulating seizure development after perinatal HI and support the hypothesis that targeting brain hyaluronidase activity may be an effective strategy to reduce seizures. Future studies are required to determine the effect of apigenin or S3 on neurohistological outcomes in the developing brain.

## 4. Materials and Methods

### 4.1. Ethics 

All procedures undertaken in this study were approved by the Animal Ethics Committee of the University of Auckland (22069, 3 May 2021) and were performed in accordance with the New Zealand Animal Welfare Act 1999 and the University of Auckland’s Code of Ethical Conduct for the use of animals for teaching and research. This study is compliant with the ARRIVE (Animal Research: Reporting of In Vivo Experiments) guidelines for reported animal research [65]. 

### 4.2. Animals and Surgical Preparations

Twenty-six time-mated Romney–Suffolk crossbred fetal sheep were instrumented at 93–94 days gestation (term ~147 days gestation). Ewes were acclimatized to the laboratory for 1 week, with regular veterinary health and welfare checks. To reduce the risk of aspiration, pellet nut feed, but not water, was withdrawn 18 h before surgery, and ewes were prophylactically given oxytetracycline (20 mg/kg; SVS Veterinary Supplies, Hamilton, New Zealand). Intravenous (i.v.) injection of propofol (5 mg/kg; Onelink, Auckland, New Zealand) was used to induce anesthesia, and following endotracheal intubation, general anesthesia was maintained using 2–3% isoflurane (Medsource, Ashburton, New Zealand) in oxygen. Ewes received a constant isotonic saline drip (250 mL/h) to maintain fluid balance. The depth of anesthesia, maternal heart rate, blood pressure, and respiration were monitored by trained anesthetic staff. 

Using aseptic techniques, a midline incision was made to expose the uterus and the fetus was partially exteriorized for instrumentation. Polyvinyl catheters (made in-house using polyvinyl tubing from SteriHealth, Dandenong South, VIC, Australia; internal diameter (ID): 0.5 mm, outer diameter (OD): 0.9 mm) were placed into the left and right brachial arteries for blood pressure measurement and preductal blood sampling. To measure amniotic fluid pressure, an additional catheter (ID: 0.8 mm, OD: 1.2 mm) was placed in the amniotic sac. To record the fetal electrocardiogram (ECG), electrodes (AS633-3SSF; Cooner Wire Company, Chatsworth, CA, USA) were subcutaneously placed over the right shoulder and at the level of the left fifth intercostal space. To measure fetal EEG activity, two pairs of electrodes (AS633-7SSF; Cooner Wire Company) were placed on the dura over the parasagittal parietal cortex bilaterally (10 and 15 mm anterior, 5 mm lateral to bregma), with a reference electrode placed over the occiput. For drug infusion, an intracerebroventricular (ICV) catheter was placed in the left lateral cerebral ventricle (6 mm anterior and 4 mm lateral to bregma) to a depth of 1.0 cm. Inflatable carotid occluder cuffs were fixed around both carotid arteries and the vertebral–occipital anastomoses were ligated [25,26].

On completion of the surgical procedures, the fetus was returned to the uterus and any lost amniotic fluid was replaced with ~500 mL sterile isotonic saline (warmed to ~39.5 °C). Antibiotics (80 mg gentamicin; Pfizer, Auckland, New Zealand) were administered into the amniotic sac and the uterus closed. The maternal laparotomy skin incision was infiltrated with a local analgesic (10 mL 0.5% bupivacaine plus adrenaline; AstraZeneca Limited, Auckland, New Zealand) for analgesia. All polyvinyl catheters and electrode leads were exteriorized via the maternal flank. Finally, to provide access for postoperative maternal care and euthanasia, a polyvinyl catheter (ID: 0.8 mm, OD: 1.2 mm) was placed in the maternal saphenous vein.

### 4.3. Postoperative Care

Following surgery, animals were housed together in individual metabolic cages with access to water and food (concentrated pelleted food; Dunstan Nutrition, Hamilton, New Zealand) ad libitum. Animal housing facilities were temperature-controlled (16 ± 1 °C, humidity 50 ± 10%) and operated on a 12 h light/dark cycle. A period of 4–5 days recovery was allowed before commencement of experiments. During the postoperative period prior to experiments, all ewes were given i.v. antibiotics, including gentamicin (40 mg for 3 days; Pfizer) and benzylpenicillin sodium (600 mg for 4 days; Onelink). Fetal and maternal vascular catheters were maintained patent by continuous infusion of heparinized saline (20 U/mL at rate of 0.15–0.20 mL/h). Fetal wellbeing was assessed through daily fetal arterial blood sampling to measure preductal pH, blood gas, base excess (ABL800 Flex analyzer; Radiometer, Auckland, New Zealand), and glucose and lactate (YSI model 2300; YSI Life Sciences, Yellow Springs, OH, USA). 

### 4.4. Experimental Recordings

All physiological recordings were started 24 h before bilateral carotid occlusion and continued for 3 days after occlusion by computer using custom data acquisition software (LabVIEW for Windows 2020; National Instruments, Austin, TX, USA). Data included fetal MAP (Novatrans II transducers, MX860; Medex, Hilliard, OH, USA), FHR derived from the ECG, and EEG. Fetal MAP was corrected for maternal movement by subtraction of amniotic fluid pressure. MAP signals were filtered with an analog fifth-order lowpass Butterworth filter with a cutoff frequency at 20 Hz, then digitized at a sampling rate of 512 Hz. The raw ECG signal was filtered with an analog first-order highpass filter with a cutoff frequency of 0.05 Hz and an analog fifth-order lowpass Bessel filter with a cutoff at 100 Hz, and then digitized at a sampling rate of 1024 Hz. RR intervals were extracted from this signal to calculate FHR. EEG signals were amplified 10,000× and processed with an analog first-order highpass filter (cutoff at 1.6 Hz) and an analog fifth order lowpass Butterworth filter (cutoff at 500 Hz), and then digitized at 4096 Hz. The EEG signal was then filtered using a lowpass filter with a digital IIR Type 2 Chebyshev filter (cutoff frequency of 120 Hz) and decimated down to 256 Hz for analysis of the raw EEG waveforms for seizures. Total EEG power (µV^2^) was calculated on the power spectrum between 1 and 20 Hz, and log transformed for presentation (decibels (dB), 10 × log10 (power)). The SEF was calculated as the frequency below which 90% of EEG power was present within the 1–20 Hz band [66].

### 4.5. Experimental Protocol

At 98–99 days of gestation, animals were randomly assigned to either sham ischemia (*n* = 9), ischemia vehicle (*n* = 8), or ischemia apigenin (*n* = 6) groups. To further explore the role of hyaluronidase activity on seizure activity following ischemia, we undertook a pilot study using the selective hyaluronidase inhibitor S3 (ischemic S3 group; *n* = 3). Fetuses of either sex were included in the study (sham ischemia: *n* = 4 female, *n* = 5 male; ischemia vehicle: *n* = 3 female, *n* = 5 male; ischemia apigenin: *n* = 4 female, *n* = 2 male; ischemia S3: *n* = 2 female, *n* = 1 male). Cerebral ischemia was induced at 09.00 h by reversible inflation of the bilateral carotid occluder cuffs for 25 min with sterile saline. Successful occlusion was confirmed by rapid suppression of EEG power and frequency within 30 s of inflation. Sham ischemia animals received no occlusion.

ICV infusions were performed using a CMA-100 microinjection pump (Carnegie Medicin; Torshamnsgatan, Sweden). Apigenin-treated fetuses received a slow bolus infusion of apigenin (0.189 mg/mL dissolved in sterile endotoxin-free modified artificial cerebrospinal fluid (aCSF) plus 0.5% dimethyl sulfoxide solution (DMSO; Sigma-Aldrich, Auckland, New Zealand)) or S3 (0.014 mg/mL dissolved in aCSF plus 0.5% DMSO) into the CSF at a rate of 1 mL/h commencing immediately after the end of ischemia for 1 h. Based on a ventricular CSF volume of approximately 6 mL and CSF clearance of approximately 50 µL/min in preterm fetal sheep [67], and assuming drug is removed with secreted CSF, this would result in an approximately 50 µM apigenin and 2 µM S3 CSF concentrations, respectively, with 0.036% DMSO. Thereafter, fetuses received a continuous infusion of apigenin (1.89 mg/mL in sterile aCSF + 5% DMSO) or S3 (0.14 mg/mL in sterile aCSF + 5% DMSO) at a rate of 42 µL/h from 1 to 24 h after the end of ischemia to maintain the 50 µM apigenin or 2 µM S3 CSF concentrations. The sham ischemia and ischemia vehicle animals received infusion of the vehicle alone (0.5% DMSO in aCSF; final CSF concentration of 0.036%) using the same infusion protocols.

Fetal arterial blood samples were collected for measurement of pH, blood gases, and glucose and lactate values at 1 h before sham ischemia/ischemia (baseline) and at 10 min, 30 min, 1 h, 2 h, 4 h, 24 h, 48 h, and 72 h after sham ischemia/ischemia. At 3 days after sham ischemia/ischemia, fetuses and ewes were killed by i.v. overdose sodium pentobarbital to the ewe (9 g Pentobarb 300; Chemstock International, Christchurch, New Zealand). Fetal brain and body weights were measured postmortem. Placement of the ICV catheter was verified postmortem and on gross histological examination.

### 4.6. Data Acquisition and Statistical Analysis

Analysis of all physiological data was performed using custom analysis program (LabVIEW for Windows). The baseline period for the analysis of all physiological data was taken as the mean of the 24 h period before carotid artery occlusion. All physiological data following sham ischemia/ischemia were assessed as hourly averages. EEG power is displayed as change in power from baseline (ΔEEG power, dB), and SEF is represented as percentage change in frequency (%SEF; Hz). Loss of signal from one fetus in the ischemia-S3 group prevented recording of MAP between 48 and 72 h following ischemia. Quantitative EEG measurements for each waveform were performed to quantify power in the delta (1.25–4.0 Hz), theta (4.25–8.0 Hz), alpha (8.25–13.0 Hz), and beta (13.25–22.0 Hz) frequency bands. The power spectra were calculated using fast Fourier transform of the continuous raw EEG recording on sequential epochs, using a 10 s Hanning window to minimize spectral leakage. The sum of the power spectrum from 1.1 to 22 Hz is the total power of the whole band. The relative power of each band was calculated as the power of each band divided by the total power and presented as a percentage.

Each minute of the raw EEG recording was analyzed manually for the presence of seizures by a single assessor (K.H.C.T.) blinded to the groups, using a custom program allowing for visualization of EEG records at a resolution of 2 s (LabVIEW for Windows). Seizures were defined as the concurrent appearance of sudden, repetitive, and rhythmic waveforms in the EEG signal lasting >10 s with a stereotypic evolving nature [38], and a minimum seizure amplitude of 20 μV. This definition ensured that seizures were easily distinguished from background activity [38] (see Figure 2 for a representative example). For each animal, we calculated the time of seizure onset and resolution of these high-amplitude seizures, the total seizure period (i.e., from first to last seizure), the total number of seizures, the total seizure time, the average maximum amplitude of all seizures, the average duration of individual seizures, the number of seizures per hour, and the seizure burden (i.e., minutes seizing per hour). A seizure was assigned to a specific minute if the seizure started in that minute. 

Statistical analyses were performed with SPSS v25 (IBM, Armonk, NY, USA). The time course of changes in cardiovascular and electrophysiological parameters were analyzed using analysis of variance (ANOVA) with time as a repeated measure. Hourly averages were used to evaluate temporal changes and analyzed in 6 h bins for the first 24 h of recovery and 24 h bins thereafter. Statistical analysis of MAP was determined using normalized data because of differences between the groups at baseline. Physiological data for the baseline and recovery periods were analyzed separately. Post-hoc comparisons were made using the Fisher’s protected least significant difference (LSD) post-hoc test when a significant effect of group or an interaction between group and time was found. Fetal biochemical data were evaluated using one-way ANOVA, followed by Fisher’s protected LSD post-hoc test when a significant overall effect was found. Analysis of fetal sex and the number of animals that developed seizures were evaluated using Pearson’s chi squared test. Seizure parameters between the ischemia groups were evaluated using nonparametric Mann–Whitney U test analysis. The number and burden of seizures per hour were analyzed using ANOVA with time as a repeated measure. Hourly averages were used to evaluate temporal changes and analyzed in 6 h bins for the first 24 h of recovery and 24 h bins thereafter. If a significant overall effect was found, the Fisher’s protected LSD post-hoc test was used to compare groups. Statistical significance was accepted when *p* < 0.05. Data are presented as mean ± SEM. 

## Figures and Tables

**Table 1:** Fetal blood gases, pH, and metabolites during the baseline, ischemia, and recovery periods.

Animal Group	Baseline	+10 min	+30 min	+1 h	+2 h	+24 h	+48 h	+72 h
*pH*								
Sham ischemia	7.38 ± 0.01	7.36 ± 0.00	7.37 ± 0.00	7.37 ± 0.00	7.37 ± 0.01	7.35 ± 0.02	7.37 ± 0.01	7.38 ± 0.01
Ischemia vehicle	7.37 ± 0.00	7.34 ± 0.00	7.37 ± 0.01	7.38 ± 0.00	7.38 ± 0.01	7.35 ± 0.01	7.36 ± 0.01	7.36 ± 0.01
Ischemia apigenin	7.35 ± 0.01 *	7.33 ± 0.01 *	7.36 ± 0.01	7.37 ± 0.01	7.38 ± 0.01	7.34 ± 0.01	7.34 ± 0.01	7.36 ± 0.01
Ischemia S3	7.38 ± 0.01	7.37 ± 0.02	7.38 ± 0.01	7.39 ± 0.01	7.39 ± 0.01	7.37 ± 0.01	7.37 ± 0.01	7.36 ± 0.01
*PaCO_2_ (mmHg)*								
Sham ischemia	49.4 ± 1.4	46.4 ± 1.2	49.3 ± 0.5	47.6 ± 0.3	48.4 ± 0.2	47.6 ± 3.3	46.1 ± 1.8	47.7 ± 1.2
Ischemia vehicle	47.5 ± 1.0	48.6 ± 1.2	46.3 ± 1.2	47.1 ± 0.8	46.5 ± 1.4	49.0 ± 1.3	47.1 ± 1.0	44.5 ± 2.5
Ischemia apigenin	50.1 ± 1.1	50.2 ± 1.2	47.9 ± 0.8	48.1 ± 1.0	47.8 ± 0.7	50.7 ± 0.7	49.2 ± 0.9	48.8 ± 1.0
Ischemia S3	47.4 ± 1.0	47.3 ± 1.7	47.3 ± 1.2	47.7 ± 1.1	47.7 ± 0.4	48.6 ± 0.6	45.5 ± 2.3	44.2 ± 2.8
*PaO_2_ (mmHg)*								
Sham ischemia	23.0 ± 1.0	26.0 ± 0.2	25.2 ± 1.1	26.8 ± 0.5	25.8 ± 0.3	25.0 ± 2.4	25.1 ± 0.4	25.2 ± 0.8
Ischemia vehicle	24.8 ± 1.1	26.9 ± 1.3	26.0 ± 0.9	26.0 ± 1.0	24.5 ± 1.3	24.8 ± 2.0	25.7 ± 1.4	27.0 ± 0.9
Ischemia apigenin	27.5 ± 1.5 *	28.5 ± 1.5	27.7 ± 1.0	27.4 ± 1.2	26.8 ± 0.8	27.9 ± 1.5	27.6 ± 1.9	27.9 ± 1.7
Ischemia S3	24.1 ± 0.6	26.4 ± 0.3	24.8 ± 0.3	24.4 ± 0.6	24.1 ± 0.2	24.3 ± 0.7	25.4 ± 0.9	26.5 ± 1.1
*Glucose (mmol/L)*								
Sham ischemia	0.9 ± 0.1	0.9 ± 0.0	1.1 ± 0.1	1.0 ± 0.1	0.9 ± 0.2	0.8 ± 0.0	0.9 ± 0.2	0.9 ± 0.1
Ischemia vehicle	1.0 ± 0.0	1.3 ± 0.1 *	1.3 ± 0.1	1.3 ± 0.1	1.2 ± 0.1	1.0 ± 0.1	1.0 ± 0.0	1.2 ± 0.1
Ischemia apigenin	1.1 ± 0.1	1.3 ± 0.1 *	1.3 ± 0.1	1.3 ± 0.1	1.3 ± 0.1	1.1 ± 0.1	0.9 ± 0.0	1.0 ± 0.2
Ischemia S3	0.8 ± 0.0	1.1 ± 0.2	1.3 ± 0.1	1.2 ± 0.1	1.2 ± 0.1	1.0 ± 0.1	0.9 ± 0.0	0.8 ± 0.0
*Lactate (mmol/L)*								
Sham ischemia	0.8 ± 0.0	0.9 ± 0.1	1.1 ± 0.1	1.0 ± 0.1	1.0 ± 0.0	1.1 ± 0.1	0.9 ± 0.1	0.8 ± 0.1
Ischemia vehicle	0.8 ± 0.1	1.9 ± 0.2 *	1.6 ± 0.1 *	1.1 ± 0.1	1.0 ± 0.1	0.8 ± 0.1	0.8 ± 0.1	0.7 ± 0.1
Ischemia apigenin	0.8 ± 0.1	2.1 ± 0.2 *	1.8 ± 0.2 *	1.3 ± 0.2	1.1 ± 0.2	0.8 ± 0.1	0.8 ± 0.0	0.8 ± 0.1
Ischemia S3	0.9 ± 0.2	2.0 ± 0.2 *	1.8 ± 0.2 *	1.4 ± 0.1	1.2 ± 0.1	1.1 ± 0.1	1.1 ± 0.1	1.0 ± 0.1

Fetal arterial blood samples were taken from the sham ischemia, ischemia vehicle, ischemia apigenin, and ischemia S3 fetuses at 60 min before carotid occlusion (baseline), and at 10 min, 30 min, 1 h, 2 h, 24 h, 48 h, and 72 h after occlusion. PaCO_2_: arterial pressure of carbon dioxide; PaO_2_: arterial pressure of oxygen. Data are presented as mean ± standard error of the mean (SEM). Between group comparisons made using one-way analysis of variance (ANOVA) and least significant difference (LSD) post-hoc test. * *p* < 0.05 vs. sham ischemia.

**Table 2:** Fetal biometric parameters.

Group	N	Female/Male	Body Weight (g)	Brain Weight (g)
Sham ischemia	9	4/5	1061.8 ± 15.8	23.4 ± 0.5
Ischemia vehicle	8	3/5	1080.0 ± 24.8	20.4 ± 0.3 *
Ischemia apigenin	6	4/2	1066.4 ± 30.5	19.5 ± 0.5 ^#^
Ischemia S3	3	2/1	1072.9 ± 45.3	18.3 ± 1.0 ^φ^

Data presented as mean ± SEM. Group comparisons made using one-way ANOVA. * *p* = 0.036; ischemia vehicle vs. sham ischemia, ^#^ *p* = 0.007; ischemia apigenin vs. sham ischemia, ^φ^ *p* = 0.002; ischemia S3 vs. sham ischemia.

**Table 3:** Characteristics of post-ischemic seizures in the ischemia groups.

Group	No. Fetuses That Developed Seizures/No. Fetuses in Group	Seizure Onset (h) ^a^	Seizure End (h) ^a^	Total Seizure Period (h) ^a^	No. of Seizures over 72 h	Total Seizure Time (min)	Mean Max. Amplitude (µV) ^a^	Mean Duration (min) ^a^
Ischemia vehicle	8/8	7.0 ± 1.0	22.8 ± 5.5	15.9 ± 5.6	23.6 ± 4.6	30.0 ± 5.9	180.3 ± 30.0	1.3 ± 0.1
Ischemia apigenin	3/6 *	5.0 ± 0.9	10.7 ± 0.3 *	5.8 ± 0.9	11.3 ± 6.4 ^φ^	10.9 ± 6.2 ^#^	175.3 ± 21.2	1.0 ± 0.0 ^ⴕ^
Ischemia S3	1/3 *	[4.7] ^b^	[15.9] ^b^	[11.2] ^b^	4.3 ± 4.2 ^#^	4.3 ± 4.0 ^#^	[239.7] ^b^	[0.9] ^b^

^a^ Note that the timing of the onset and end of seizures, the total seizure period (i.e., from first to last seizure), maximum seizure amplitude, and average seizure duration data are from fetuses that developed seizures (i.e., ischemia vehicle (*n* = 8), ischemia apigenin (*n* = 3), ischemia S3 (*n* = 1)). ^b^ The seizure data from the one animal that exhibited seizures in the ischemia S3 group are presented. Data are presented as mean ± SEM. Statistical analysis of the number of animals that developed seizures was evaluated using Pearson’s chi squared test. * *p* < 0.05 vs. ischemia vehicle. Other seizure parameters were evaluated using the Mann–Whitney U test. ^#^ *p* < 0.05 vs. ischemia vehicle. ^φ^ *p* = 0.07, ischemia apigenin vs. ischemia vehicle. ^ⴕ^ *p* = 0.08, ischemia apigenin vs. ischemia vehicle.

## Data Availability

The data presented in this study are available on request from the corresponding author.

## Supplementary Materials

The following supporting information can be downloaded at: https://www.mdpi.com/article/10.3390/ijms242316926/s1.
