# Supplementary material for: Postischemic Infusion of Apigenin Reduces Seizure Burden in Preterm Fetal Sheep
Source: Int J Mol Sci. 2023 Nov 29;24(23):16926. doi: 10.3390/ijms242316926 (PMC10706648; doi:10.3390/ijms242316926)
Supplement: Supplementary file 1 [file ijms-24-16926-s001.zip › ijms-2682013-supplementary.pdf]

Supplementary Materials

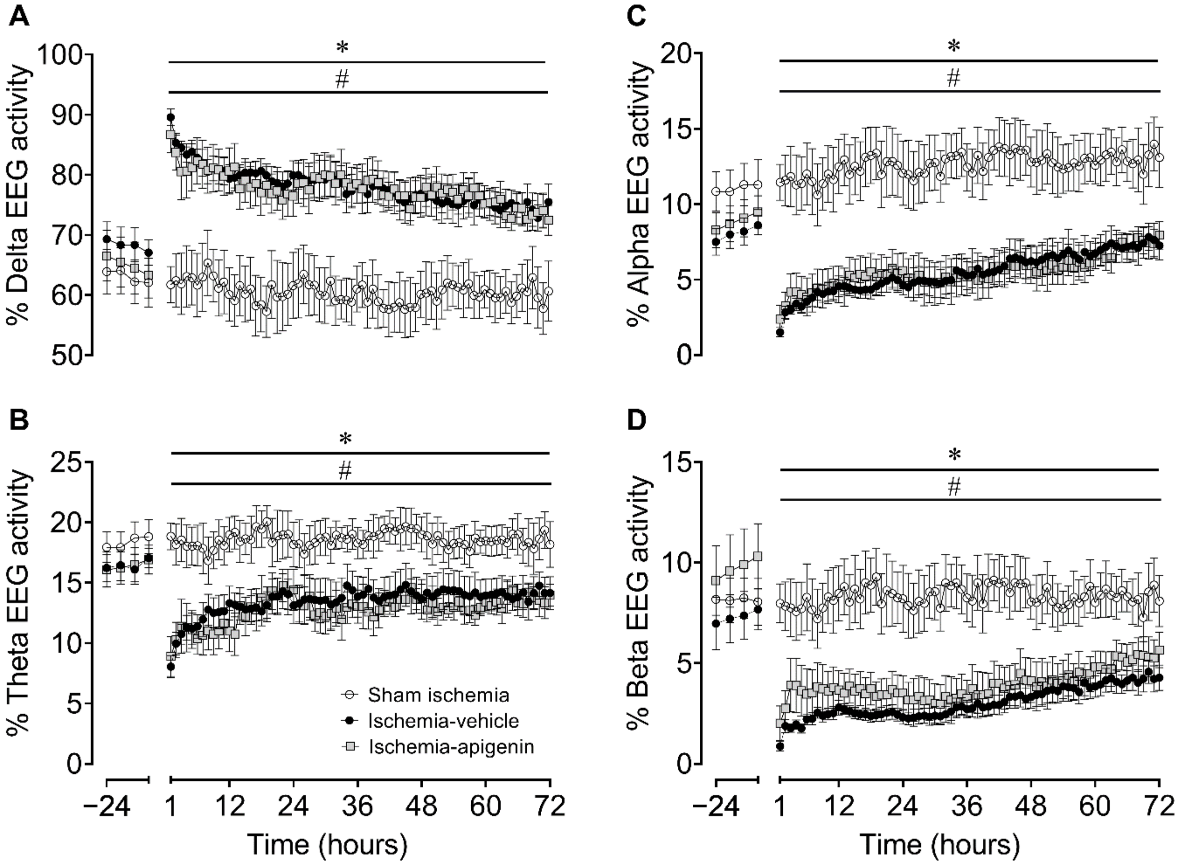

Figure S1. Time sequence of changes in the percentage of fetal electroencephalographic (EEG) power in the delta- (**A**), theta- (**B**), alpha- (**C**), and beta- (**D**) frequency bands from 24 h before until 72 h after carotid occlusion in the sham ischemia (open circles; n = 9, 4 females, 5 males), ischemia-vehicle (closed circles; n = 8, 3 females, 5 males), and ischemia-apigenin (grey squares; n = 6, 4 females, 2 males) groups. Time zero denotes the end of the period of 25 min of cerebral ischemia (not shown). Note that for presentation purposes, EEG datasets for ischemia-apigenin and ischemia-S3 (see Fig. S2) are plotted as separate graphs, but contain the same sham ischemia and ischemia-vehicle datasets. Data are mean ± standard error of the mean (SEM) (of 6-h averages for baseline period, and 1-h averages post-occlusion). Statistical significance was determined by repeated-measures analysis of variance (ANOVA), followed by Fisher's least significant difference (LSD) post hoc analysis. *$P<0.05$, sham ischemia vs. ischemia-vehicle; #$P<0.05$, sham ischemia vs. ischemia-apigenin.

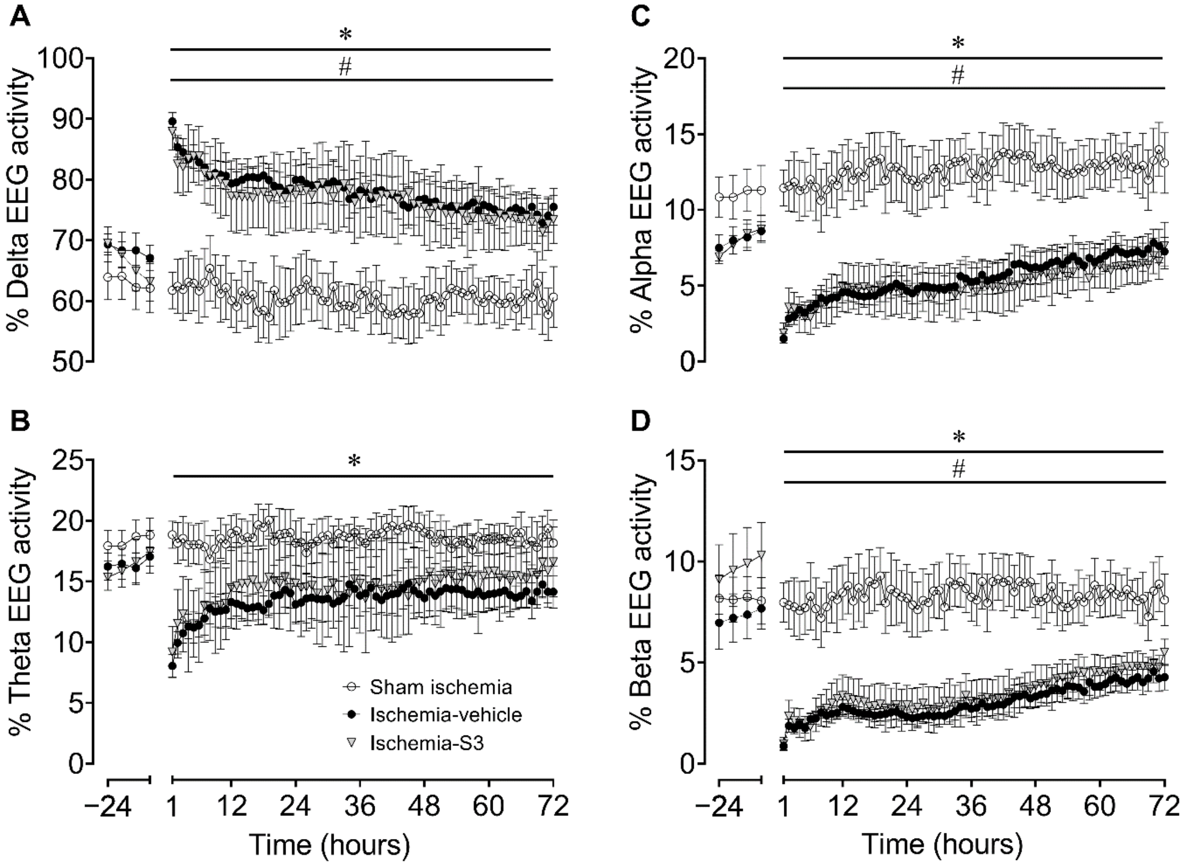

Figure S2. Time sequence of changes in the percentage of EEG power in the delta- (**A**), theta- (**B**), alpha- (**C**), and beta- (**D**) frequency bands from 24 h before until 72 h after carotid occlusion in the sham ischemia (open circles; n = 9, 4 females, 5 males), ischemia-vehicle (closed circles; n = 8, 3 females, 5 males), and ischemia-S3 (C, grey triangles; n = 3, 2 females, 1 male) groups. Time zero denotes the end of the period of 25 min of cerebral ischemia (not shown). Note that for presentation purposes, EEG datasets for ischemia-apigenin (see Supplementary Fig. 1) and ischemia-S3 are plotted as separate graphs, but contain the same sham ischemia and ischemia-vehicle datasets. Data are mean ± SEM (of 6-h averages for baseline period, and 1-h averages post-occlusion). Statistical significance was determined by repeated-measures ANOVA, followed by Fisher's LSD post hoc analysis. *P<0.05, sham ischemia vs. ischemia-vehicle; #P<0.05, sham ischemia vs. ischemia-S3.
